# CRISPR/Cas9-Mediated Mutagenesis of *BrLEAFY* Delays the Bolting Time in Chinese Cabbage (*Brassica rapa* L. ssp. *pekinensis*)

**DOI:** 10.3390/ijms24010541

**Published:** 2022-12-29

**Authors:** Yun-Hee Shin, Young-Doo Park

**Affiliations:** Department of Horticultural Biotechnology, Kyung Hee University, 1732 Deogyoung-daero, Giheung-gu, Yongin-si 17104, Gyeonggi-do, Republic of Korea

**Keywords:** *Brassica rapa*, CRISPR/Cas9, floral identity, late bolting, *LEAFY* gene

## Abstract

Chinese cabbage has unintended bolting in early spring due to sudden climate change. In this study, late-bolting Chinese cabbage lines were developed via mutagenesis of the *BrLEAFY* (*BrLFY*) gene, a transcription factor that determines floral identity, using the clustered regularly interspaced short palindromic repeat (CRISPR)/CRISPR-associated protein 9 (CRISPR/Cas9) system. Double-strand break of the target region via gene editing based on nonhomologous end joining (NHEJ) was applied to acquire useful traits in plants. Based on the ‘CT001’ pseudomolecule, a single guide RNA (sgRNA) was designed and the gene-editing vector was constructed. *Agrobacterium*-mediated transformation was used to generate a Chinese cabbage line in which the sequence of the *BrLFY* paralogs was edited. In particular, single base inserted mutations occurred in the *BrLFY* paralogs of the LFY-7 and LFY-13 lines, and one copy of T-DNA was inserted into the intergenic region. The selected *LFY*-edited lines displayed continuous vegetative growth and late bolting compared to the control inbred line, ‘CT001’. Further, some *LFY*-edited lines showing late bolting were advanced to the next generation. The T-DNA-free E_1_ *LFY*-edited lines bolted later than the inbred line, ‘CT001’. Overall, CRISPR/Cas9-mediated mutagenesis of the *BrLFY* gene was found to delay the bolting time. Accordingly, CRISPR/Cas9 is considered an available method for the molecular breeding of crops.

## 1. Introduction

In Chinese cabbage, bolting, which appears as flower buds from leaves, is controlled by internal signals and environmental factors. Internal signals have been investigated in various pathways, including autonomous circadian rhythm and floral meristem identity [1,2,3]. In particular, the transcription factors (TFs), *LEAFY* (*LFY*), *APETALA1* (*AP1*), and *CAULIFLOWER* (*CAL*), and the floral meristem identity pathway control flower development and flowering time [4,5]. Functional analysis of these genes was mainly performed to determine the morphology of flower organs and assess meristem formation. For example, *lfy/ap* double mutants showed complete transformation of all whorled flowers into inflorescence [6]. Floral meristem identity genes interact together to result in morphologic constructions, including short internodes and suppression of axillary buds.

Temperature is a typical environmental factor that induces bolting. Chinese cabbage is a seed vernalization-type plant. After a certain period of response to low temperatures, and the external temperature suitable for growth increases, Chinese cabbage displays bolting and flowering [7]. Vernalization is usually necessary to promote flowering; however, recent rapid changes in temperatures due to climate change have sometimes led to undesirable premature bolting, which affects sufficient vegetative growth for leaf formation and causes commercial quality degradation in vegetable crops [8,9]. Premature bolting and flowering of leafy plants like lettuce, radish, and Chinese cabbage are unfavorable agricultural traits, resulting in significant economic loss. Chinese cabbage is a leafy vegetable that is widely produced all over the world. Chinese cabbage, the main ingredient of kimchi, consumes 2.33 million tons per year in South Korea. The storage period of Chinese cabbage is 3 to 4 months at the longest, and because freshness is required, production is equal to consumption. Due to these characteristics, stable production is very important because the price fluctuates so much that a 10–20% decrease in production volume can increase the price by 2–3 times [10]. The yield of Chinese cabbage is decreased due to premature bolting; thus, the development and breeding of late-bolting crops are needed to address climate change [11].

Resistant crops against biotic or abiotic stresses have been bred by inserting exogenous resistance genes or regulating the expression of endogenous genes. In addition, with the development of molecular biological technology, genetic scissors that induce sequence mutations have been developed. Clustered regularly interspaced short palindromic repeat (CRISPR)/CRISPR-associated protein 9 (CRISPR/Cas9)-mediated mutagenesis of key genes in Chinese cabbage (*Brassica rapa* ssp. *pekinensis*) was performed to develop late-bolting Chinese cabbage plants. *VERNALIZATION 1* (*BrVRN1*), a DNA-binding encoding gene that suppresses *FLOWERING LOCUS C* (*FLC*), is required for the normal processes of the flowering pathway. The bolting time of the *BrVRN1* gene-edited lines was found to be delayed regardless of vernalization treatment [12]. Similarly, the *SUPPRESSOR OF OVEREXPRESSION OF CONSTANCE 1* (*BrSOC1*), flowering integrator gene, is involved in late flowering. Previously, no bolting phenotype was observed for the *BrSOC1s* mutagenesis transgenic lines within 100 days [13]. As studies on bolting and flowering analyses to target flower meristem identity genes using the CRISPR/Cas9 system are limited, more investigations need to be performed.

CRISPR/Cas9-mediated mutagenesis of the target DNA sequence involves several steps. Briefly, a target DNA sequence that includes NGG as a protospacer adjacent motif (PAM) is recognized, and double-strand breaks (DSBs) are introduced by a nuclease near the target DNA sequence. Within the cell, the process of self-repairing DSBs occurs via endogenous nonhomologous end-joining (NHEJ) or homology-directed repair (HDR) pathways [14,15].

Gene-editing technologies have been developed and are increasingly utilized [15,16]. Compared with previous generations of engineered nucleases, CRISPR/Cas9 has improved accuracy, resulting in reduced mosaic or off-target effects.

The beginning of the reproductive phase is regulated by a number of flowering pathways identified by various flowering time mutants [17]. Several mutants were engaged in the detection of environmental conditions or gene regulation, according to molecular characterization of mutants related to flowering time [18,19,20]. The meristem identity genes essential for the floral transition are finally induced as a result of the flowering process. One of the meristem identity genes, *LFY*, is expressed prior to flower emergence, and when regulated, has the most significant effect on the floral transition in *Brassica rapa* [21]. At the floral transition, *LFY* functions as a transcriptional regulator to promote the expression of a second meristem identity gene, *AP1* [18,22].

In this study, CRISPR/Cas9-mediated mutagenesis of the *BrLEAFY* (*BrLFY*) gene was observed in the *LFY*-edited lines. The *LFY*-edited lines displayed late bolting compared to the inbred line ‘CT001’. Based on the findings regarding *BrLFY* gene function using the CRISPR/Cas9 technology, late bolting can be induced in Chinese cabbage via mutation of the LFY amino acid sequence. Accordingly, the CRISPR/Cas9 system can be utilized for gene-edited lines under abiotic stress, and gene editing can be effectively applied to the development of practical crops through direct mutagenesis of the target gene sequence.

## 2. Results

### 2.1. Target Gene Structure Analysis and LFY-Editing Vector Construction

**Figure 1:**
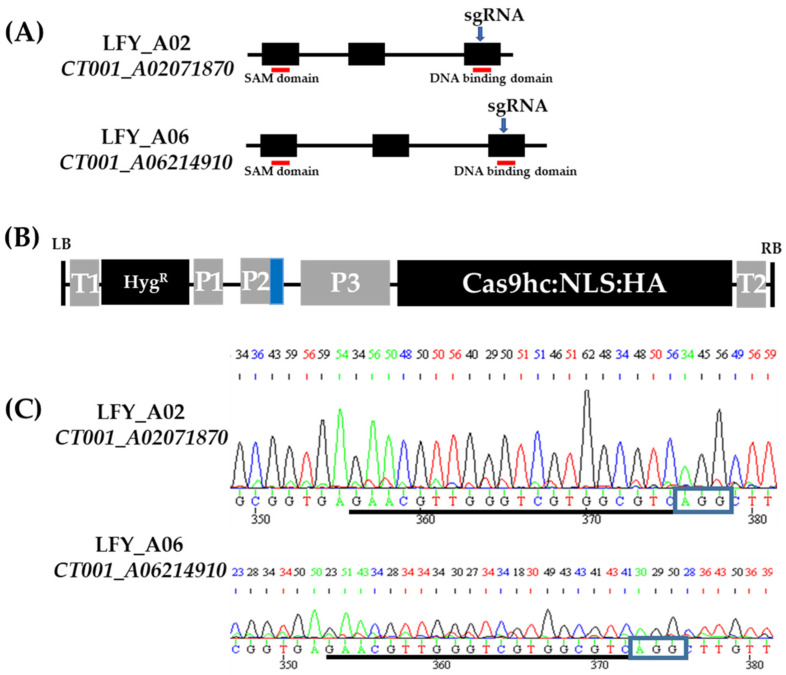
CRISPR/Cas9 gene editing vector construction targeting *BrLFY* paralogs. (**A**) Gene structure of the *BrLFY* paralogs. Black box, exon regions; Black line, intron regions; Blue arrow, target region of single guide RNA (sgRNA); and Red line, conserved domain. (**B**) Structure of CRISPR/Cas9 gene editing vector with sgRNA targeting the *BrLFY* paralogs based on pHAtC. LB, left border; T1, NOS terminator; Hyg^R^, hygromycin resistance gene; P1, NOS promoter; P2, *Arabidopsis* U6 promoter; P3, 35S promoter; Cas9hc:NLS:HA, human-codon-optimized Cas9 with the nuclear localization signal and an HA epitope; T2, 35S terminator; RB, right border; and Blue box, sgRNA, and sgRNA scaffold. (**C**) Chromatograms of single guide RNA (sgRNA) sequence in the *BrLFY* paralogs of inbred line, ‘CT001’. sgRNA sequence is underlined in black and blue rectangles indicating PAM sequence.

To construct an *LFY*-editing vector, orthologs of the *BrLFY* gene were analyzed based on the *AtLFY* (AT5G61850) coding DNA sequence using the *B*. *rapa* database (BRAD, http://brassicadb.org, accessed on 8 April 2019). Based on the similar sequences obtained, two paralogs (*CT001_A02071870* and *CT001_A06214910*) were identified in the Chinese cabbage genome. The amino acid sequence similarity between the *BrLFY* paralogs was 90%. *CT001_A02071870* was identified on the A02 chromosome while *CT001_A06214910* was identified on the A06 chromosome. Both genes are composed of three exons spanning 2.5–2.8 kb. The single guide RNA (sgRNA) was designed simultaneously with target paralogs and included a partial sequence of the exonic region containing the NGG sequence (PAM sequence). In the third exon, the DNA-binding domain of the *BrLFY* gene was selected as the target region (Figure 1). The off-targeting effect was analyzed, and an *LFY*-editing vector (pLFY) carrying sgRNA, sgRNA scaffold, and Cas9 components was constructed. pLFY was modified based on the pHAtC gene editing vector, which contained hygromycin resistance as a selection marker and transformed into *Agrobacterium tumefaciens* LBA4404.

### 2.2. Selection of Tentative LFY-Edited Chinese Cabbage Lines Using PCR Analysis

**Figure 2:**
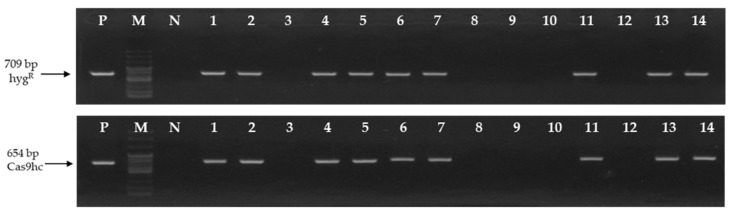
Identification of the tentative E_0_ *LFY*-edited lines by polymersase chain reaction (PCR) analysis using the Hyg^R^ and Cas9hc primer sets. The 709 bp and 654 bp PCR amplicons are indicated by an arrow, respectively. P, positive control; M, 100 bp DNA ladder; N, negative control; Numbering lane, tentative *LFY*-edited lines.

To develop the *LFY*-edited lines, seeds of the inbred line, ‘CT001’ were germinated in vitro and re-differentiation of shoots derived from hypocotyls was induced by *Agrobacterium* transformation. Fourteen independent tentative E_0_ *LFY*-edited lines were obtained. To analyze the gene mutations induced by CRISPR/Cas9, DNA was extracted from leaves to select the T-DNA-inserted lines. Polymerase chain reaction (PCR) analysis was performed using two primer sets that amplify a part of Hyg^R^ and Cas9hc:NLS:HA (Cas9hc) present in the T-DNA (Table S1 in Supplementary Materials). Nine of the 14 E_0_ lines appeared as target amplicons (Figure 2).

### 2.3. Identification of CRISPR/Cas9-Mediated Mutagenesis of the BrLFY Paralogs

**Figure 3:**
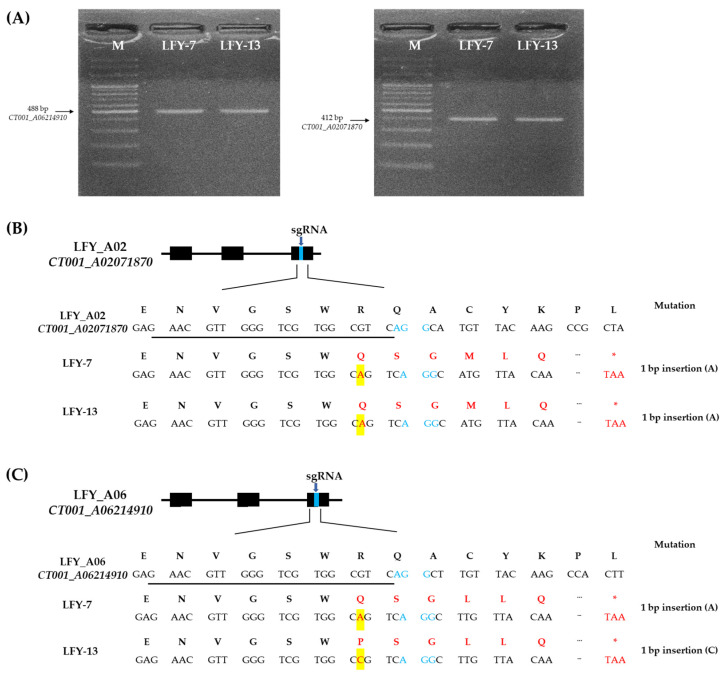
Sequence-based detection of mutations induced by CRISPR/Cas9 system in the *BrLFY* paralogs of the E_0_ *LFY*-edited lines. (**A**) PCR analysis results based on the target gene-specific amplifying primer sets of the E_0_ *LFY*-edited lines. M, 100 bp DNA ladder; Numbering lane, *LFY*-edited lines. (**B**) Mutagenesis of *CT001_A02071870* at the genomic DNA and amino acid levels in the LFY-7 and LFY-13 edited lines. (**C**) Mutagenesis of *CT001_A06214910* at the genomic DNA and amino acid levels in the LFY-7 and LFY-13 edited lines. sgRNA sequence is underlined in black and PAM sequence is shown in blue font. Nucleotide and amino acid mutations are indicated by a red font and base insertions are highlighted in yellow boxes. Termination codon is indicated in red asterisk.

Nine independent E_0_ *LFY*-edited lines with target amplicons were analyzed for target sequence mutations. As a result, four E_0_ *LFY*-edited lines showed a single base insertion in *CT001_A02071870* and three E_0_ *LFY*-edited lines showed a single base insertion in *CT001_A06214910* (Figure S1 in Supplementary Materials). Among the selected *LFY*-edited lines with a single base insertion, the E_0_ *LFY*-edited lines (LFY-7 and LFY-13) were identified again via PCR and reverse-transcript PCR (RT-PCR) analysis using target gene-specific amplification primer sets (Table S1 in Supplementary Materials; Figure 3A). Sequence confirmation of the target region in the two paralogs was performed, and insertion mutation of a single base was induced by CRISPR/Cas9 (Figure 3B). An adenine base of the target regions near the PAM sequence of sgRNA in *BrLFY* paralogs was identified in the E_0_ LFY-7 line. The E_0_ LFY-13 line was also found to have an insertion mutation in the target regions of the *BrLFY* paralogs. In the *CT001_A02071870* gene of the E_0_ LFY-13 line, a single base (adenine) was inserted 4 bp upstream of the PAM sequence. In addition, the *CT001_A06214910* gene was targeted and a cytosine base was inserted 4 bp upstream of the PAM sequence (Figure 3C). CRISPR/Cas9-mediated mutagenesis of the *BrLFY* paralogs resulted in a frameshift and premature termination codons.

Then, the expression levels of *BrLFY* paralogs in the E_0_ *LFY*-edited lines were analyzed using quantitative real-time PCR (qPCR) analysis. Overall, the expression level of *BrLFY* paralogs was lower in the E_0_ *LFY*-edited lines than in the inbred line ‘CT001’. The occurrence of insertion nucleotide mutation in the coding region affected the expression levels of *BrLFY* paralogs (Figure S2 in Supplementary Materials).

### 2.4. Observation of the Bolting Time of the Inbred Line, ‘CT001’, and E_0_ LFY-Edited Lines

**Figure 4:**
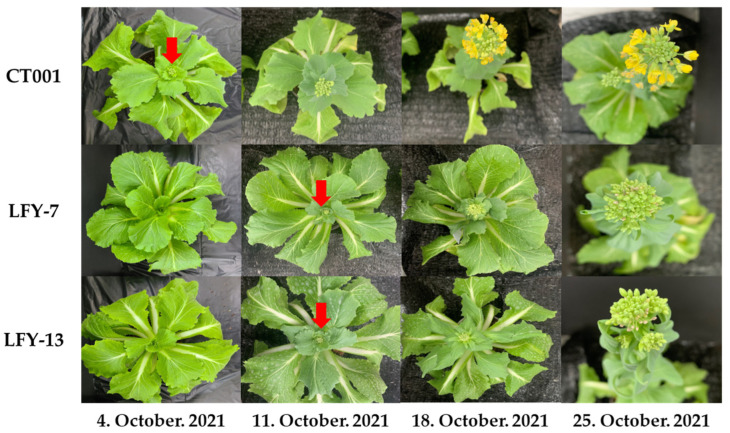
Confirmation of the bolting time and flower bud phenotype for ‘CT001’ and the E_0_ LFY-edited lines. Red arrow, emergence of the first flower bud.

To determine whether the *BrLFY* gene was related to bolting, inbred line, ‘CT001’, and E_0_ *LFY*-edited lines were planted in a pot and placed in a cold room at KyungHee Univ. After vernalization, the inbred line, ‘CT001’ and LFY-7, and LFY-13 edited lines were transferred to a greenhouse. Normal growth and development were observed for all three lines. A difference in bolting and flowering time was found between the inbred line, ‘CT001’, and *LFY*-edited lines. Further, in the initial stage, the *LFY*-edited lines had lower heights and more leaves. The first flower bud appeared in ‘CT001’. However, bolting was delayed in LFY-7 and LFY-13 lines by approximately 7 days compared to that in ‘CT001’ (Figure 4). There was no difference in the flower bud shape between ‘CT001’ and the E_0_ *LFY*-edited lines. These results indicate that the mutation of the *BrLFY* gene in the *LFY*-edited lines resulted in delayed bolting in Chinese cabbage.

### 2.5. T-DNA Copy Number and Site Analysis of the E_0_ LFY-Edited Lines

**Figure 5:**
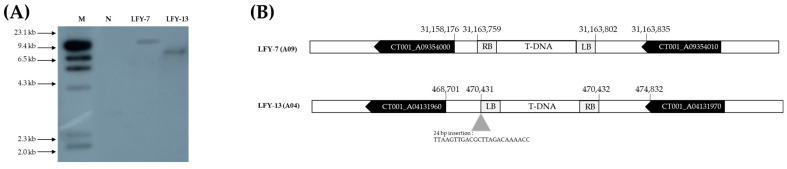
Analysis of T-DNA copy number and insertion site in the E_0_ *LFY*-edited lines. (**A**) Southern hybridization analysis results confirming the number of T-DNA copies inserted into the E_0_ *LFY*-edited line genome. Forty micrograms of genomic DNA were digested with *Eco*RI for 4 h and separated overnight on a 1.0% agarose gel at 20 V. Digested DNA was blotted onto a Hybond N^+^ nylon membrane. Probe DNA with α-^32^P-labeled dCTP was obtained, and hybridization was carried out at 65 °C in a shaking incubator. M, λ *Hin*dIII molecular ladder; N, negative control; Lane, E_0_ *LFY*-edited lines showing late-bolting. (**B**) Results of the junction region sequences of T-DNA in the E_0_ *LFY*-edited lines genome using a modified variable argument thermal asymmetric interlaced PCR (VA-TAIL PCR) analysis. T-DNA was inserted into the intergenic region of the genome of the E_0_ *LFY*-edited lines.

The T-DNA copy number was confirmed using Southern analysis, probing with the *hpt* gene. A 709 bp probe was designed and radiolabeled with ^32^P. The site was determined using the λ *Hin*dIII molecular ladder markers on the blot. Based on the results, LFY-7 and LFY-13 contained a single copy insertion (Figure 5A). No signal was observed in the negative control line, ‘CT001’. To determine whether T-DNA insertion affected other gene functions, the junction regions between the Chinese cabbage genome and T-DNA border were amplified by variable argument thermal asymmetric interlaced PCR (VA-TAIL PCR) analysis, as previously described. Using six arbitrary degenerate (AD) primers, at least one fragment was amplified in the junction region. Among the examined amplicons, two contained specific junction sequences.

T-DNA was stably inserted into the intergenic region of the LFY-7 and LFY-13 edited lines, including the insertion of some nucleotide sequences, without affecting the other gene functions (Figure 5B). In the LFY-7 edited line, T-DNA was inserted in the intergenic region of the *CT001_A09354000* and *CT001_A09354010*. T-DNA of the LFY-13 edited line was located on the intergenic region of the *CT001_A04131960* and *CT001_A04131970*.

### 2.6. Inheritance of Base Mutation in the E_1_ T-DNA-Free LFY-Edited Lines

The E_1_ *LFY*-edited lines were analyzed to identify the target sequence mutations. The T-DNA-free E_1_ *LFY*-edited lines were confirmed by PCR analysis and partial lines were selected using both primer sets (Figure S3 in Supplementary Materials). Three of the 12 LFY-7 and one of the five LFY-13 edited lines were selected. Target sequence mutation analysis was conducted using PCR analysis with the target gene-specific amplifying primer sets (Table S1 in Supplementary Materials). Base mutations induced by CRISPR/Cas9 in the two paralogs were stably inherited by the next generation. Genetic mutations were compared between E_0_ and E_1_ *LFY*-edited lines. Some E_1_ *LFY*-edited lines with T-DNA insertions retained the same mutated sequence as the E_0_ *LFY*-edited lines. However, some did not inherit mutated sequences and showed other sequence mutations. The E_1_ #7-1 line with T-DNA insertion inherited the mutation pattern of *CT001_A02071870* and *CT001_A06214910* genes, but the E_1_ #7-4 line with T-DNA insertion showed an altered mutation pattern with cytosine base inserted in the *CT001_A06214910* gene. Similarly, the E_1_ #13-3 line with T-DNA insertion showed a mutation pattern in which one base was inserted in the *CT001_A02071870* and *CT001_A06214910* genes.

**Figure 6:**
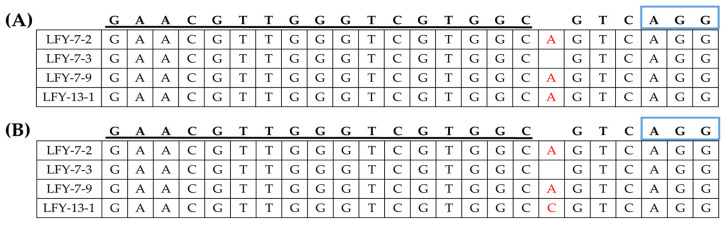
Identification of the generated mutagenesis of the *BrLFY* paralogs in the T-DNA-free E_1_ *LFY*-edited lines. (**A**) Mutagenesis of *CT001_A02071870* at the genomic DNA level in the E_1_ *LFY*-edited lines. (**B**) Mutagenesis of *CT001_A06214910* at the genomic DNA level in the E_1_ *LFY*-edited lines. sgRNA sequence is underlined in black and blue rectangles indicating PAM sequence. Insertion mutations are indicated using red font.

Among the progeny lines of LFY-7, the same base mutation pattern was found in the T-DNA-free E_1_ #7-2 and #7-9 lines (Figure 6). An adenine base was inserted in both paralogs, and owing to this mutation, a premature termination was predicted to appear. Likewise, the E_1_ #13-1 progeny line of the T-DNA-free LFY-13 line, *CT001_A02071870*, showed insertion of an adenine base, while *CT001_A06214910* showed insertion of a cytosine base.

### 2.7. Delayed Bolting in the E_1_ T-DNA-Free LFY-Edited Lines

To confirm the bolting time and observe the developmental processes, the inbred line ‘CT001’ and E_1_ T-DNA-free *LFY*-edited lines were cold-treated in a cold room at KyungHee Univ.

**Figure 7:**
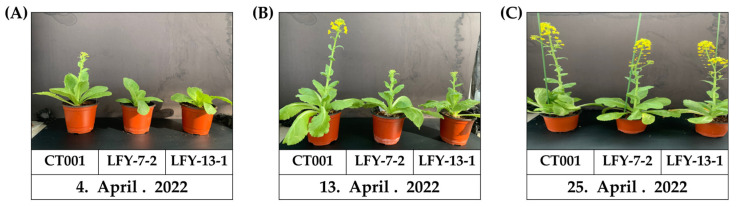
Confirmation of bolting time and normal growth of ‘CT001’ and E_1_ T-DNA-free *LFY*-edited lines. (**A**) Early stage of bolting. Bolting of the inbred line ‘CT001’ (left); (**B**) Middle stage of bolting. Bolting of the E_1_ T-DNA-free *LFY*-edited lines (middle and right); (**C**) End stage of bolting. Left, inbred line ‘CT001’; Middle and right, E_1_ T-DNA-free *LFY*-edited lines. All lines showed normal growth.

The flowering time of the E_1_ T-DNA-free *LFY*-edited lines was later than that of the control line, ‘CT001’ (Figure 7A). When bolting was observed in the inbred line, ‘CT001’, the E_1_ *LFY*-edited lines were still in the vegetative growth stage. Thereafter, when the delay was approximately 10 days on average, the E_1_ #7-2 and #13-1 *LFY*-edited lines were converted to the reproductive growth stage, and bolting was observed. The E_1_ lines displayed a phenotype similar to that of the E_0_ *LFY*-edited lines. Except for the delayed bolting phenomenon, no differences were found in the developmental processes in all lines. The delay in bolting time was confirmed to be caused by CRISPR/Cas9-mediated mutagenesis of the *BrLFY* gene.

## 3. Discussion

TFs possess reprogramming activity that enables changes from one cell type to another. The *BrLEAFY* (*BrLFY*) gene is a TF that determines flower identity and has two domains. A sterile alpha motif (SAM) is located at the N-terminal amino acid sequence of the *BrLFY* gene, while a DNA-binding domain (DBD) is located at the C-terminal amino acid sequence [23]. The DNA-binding domain is included in a protein involved in the regulation of gene expression and can recognize a specific DNA sequence or have a general affinity for DNA. The *BrLFY* gene has a unique helix–turn–helix structure observed in proteins that regulate developmental processes [24]. During the developmental processes of floral plants, the transition from vegetative to reproductive growth occurs.

The *LFY* gene directly or indirectly interacts with another floral identity gene, *APETALA1* (*AP1*) [22]. The downstream network of *LFY* controls a feed-forward reaction that regulates the upregulation of *AP1*. *LFY* is also associated with gibberellin, a hormone involved in flowering [25]. Increasing the LFY activity decreases gibberellin levels, which increases the levels of GA-GID1-DELLA proteins (DELLAs). Flowering is promoted when DELLAs are decomposed; however, owing to the decrease in gibberellin levels, DELLAs cannot be degraded, which affects the delay in flowering [26].

Previous studies revealed that the *LFY* gene acts as a switch that induces flowering. Overexpression of *LFY* induces early flowering in *Arabidopsis* transgenic plants [27]. A transgenic Chinese cabbage plant developed by RNA interference-mediated transformation displayed late bolting and flowering due to the downregulation of *LFY* gene expression [28]. Transgenic plants were found to continue to grow in the vegetative stage. Further, the number of leaves was greater than that of the control, and the plant stem length was shorter than that of the control owing to the delayed transition to reproductive growth. These studies indicate that changes in LFY levels affect flowering times.

The *BrLFY* transcript was found to be present in all Chinese cabbage tissues. It was primarily expressed in young leaves, moderately in older leaves, and slightly in roots and flowers [21]. Similarly, in this study, the expression of the *BrLFY* paralogs was confirmed in tissues such as leaves, flowers, stems, and flower buds of the inbred line ‘CT001’ (Figure S4 in Supplementary Materials). In comparison to the shoot apical meristem and other tissues in Arabidopsis, the *LFY* gene expression was higher in young and mature leaves [29]. In *Brassica juncea*, mature leaves showed the highest level of *BjLFY* expression, followed by stems and bracts [30]. In contrast, it was observed that the *AFL1* gene of apple and the *FaLFY* gene of strawberry were not expressed in leaves [31,32].

It is known that the *LFY* gene is involved in flowering by interacting with other genes. *FLOWERING LOCUS T* (*FT*) expression directly promotes flowering by regulating and inducing the expression of *AP1* and *LFY* [33]. Overexpression of *AGAMOUS-LIKE 17* (*AGL17*) significantly increased the expression levels of *CONSTANS* (*CO*), *LFY*, and *SOC1* and induced early flowering [34]. The B-functional proteins have direct and functional interaction with *AP1*, *LFY*, and *UNUSUAL FLORAL ORGANS* (*UFO*), as shown by the analysis of the protein binding network between the BrMADS-box proteins [35].

Gene editing studies have been performed using gene scissors that were developed in various forms. Gene editing has been used in research for more than 20 years and has developed rapidly. Research on gene editing began with ZFN, in which Fok1 performs cutting by recognizing a specific DNA sequence using a protein that is structurally stabilized by zinc ions [36]. Since then, Talen, which has improved accuracy owing to the precise recognition of DNA bases, has been developed as a second-generation gene scissor [37]. As a defense mechanism in bacteria, when a virus invades, a DNA fragment is inserted into its genome. A small RNA was prepared and bound to a Cas9 protein. When the virus re-invades, RNA finds and binds to the viral DNA, which is then cut by Cas9 and blocked from replicating [38]. By applying this principle to plants, changes in the nucleotide sequence of a specific gene can be generated. Gene editing studies have been conducted for various animals and plants [39].

Multiple sgRNAs and promoters were configured in one vector to induce the mutagenesis of several homologous genes simultaneously. A single CRISPR/Cas9 editing vector that can edit the *ZmTGA* family was constructed to generate knockout mutants. To induce the simultaneous mutation of the three homologous genes, two targets were selected based on the conserved sequence [40]. The *ZmTGA9* triple mutants failed to produce pollen grains. Homologous genes must be simultaneously targeted to analyze gene function, and an effective sgRNA must be designed.

Using conserved sequences, two to five homologous genes can be targeted using a single sgRNA sequence. The *phytoene desaturase* (*PDS*) gene involved in carotenoid biosynthesis was analyzed in Chinese kale (*Brassica oleracea* var. *alboglabra*). The CRISPR/Cas9 system was found to generate mutations in two homologous *PDS* genes using the one sgRNA, which targets the conserved sequence of homologous genes. *BaPDS1* and *BaPDS2* double mutants at the target site exhibit a clear albino phenotype [41]. *Squamosa-promoter binding protein-like 3* (*SPL3*), a gene predicted to influence the regulation of developmental stage transition, was found to have five homologous genes in the rapeseed (*brassica napus*) genome. CRISPR/Cas9-mediated mutations were induced by selecting one sgRNA using the consensus sequence of five *SPL3* homologous genes. Two individual lines with sequence mutations at all five target sites were identified, and *BnSPL3* mutants displayed a developmentally delayed phenotype [42]. These results indicate that one conserved sgRNA was efficient for mutagenesis. In this study, sgRNAs were designed to target *BrLFY* paralogs using conserved sequences (Figure 1). Further, sgRNA was designed to target the exonic regions of the *BrLFY* genes identified to have two copies in the target genome.

CRISPR/Cas9-mediated mutagenesis occurred in the target regions of both *BrLFY* paralogs (*CT001_A02071870* and *CT001_A06214910*). In this study, mutagenesis of *CT001_A02071870* and *CT001_A06214910* was performed at the genomic and amino acid levels in the LFY-7 and LFY-13 edited lines. A frameshift was predicted to occur because of the confirmation of one base insertion, which resulted in a change in the amino acid sequence and an early termination codon (Figure 3). Accordingly, normal LFY protein production was not performed, resulting in delayed bolting relative to that in the inbred line, ‘CT001’. Similarly, in plants with one nucleotide inserted into the target sequence of the bolting-related *VERNALIZATION1* (*VRN1*) gene of Chinese cabbage, bolting and flowering were delayed owing to loss-of-function of the *VRN1* gene [12].

The position of the inserted T-DNA in the intergenic region, which does not affect the expression levels of other genes, was confirmed. Subsequently, transgene isolation was carried out using advanced generation progresses. The T-DNA-free E_1_ *LFY*-edited lines bolted later than the inbred line, ‘CT001’, indicating stable maintenance of CRISPR/Cas9-mediated target sequence mutation.

Further, gene editing technology is identified to be distinct from genetically modified organisms (GMOs) as artificial genes are not injected into the genomes of animals and plants, and the existing genes of animals and plants are reconstructed by themselves [43,44]. With the advancement and success of gene editing technology, it is possible to minimize side effects caused by reconstructing the nucleotide sequence via the insertion or deletion of DNA in the base sequence of a specific gene.

Most of the functional analysis of the *LFY* gene was limited to flower organs, but this research derived novel findings by conducting a study on the bolting phenomenon. Our data also showed that the CRISPR/Cas9 system is effective, and CRISPR/Cas9-mediated mutagenesis of *BrLEAFY* delays the bolting time in Chinese cabbage (*Brassica rapa* L. ssp. *pekinensis*).

## 4. Materials and Methods

### 4.1. Design of Efficient sgRNA and Construction of the Gene Editing Vector

The orthologs of the *BrLFY* gene were analyzed using the full-length *AtLFY* (AT5G61850) gene coding DNA sequence from the Arabidopsis Information Resource (TAIR) database (TAIR, https://www.arabidopsis.org/index.jsp, accessed on 8 April 2019). The *BrLFY* gene sequence was confirmed by matching the obtained sequence to the *Brassica rapa* genome (BRAD, http://brassicadb.org, accessed on 8 April 2019). Two paralogs of *BrLFY* (*CT001_A02071870* and *CT001_A06214910*) were present in the genome of the inbred line, ‘CT001’. The design of efficient sgRNA sequences and construction of gene editing vector was performed as described previously [45].

sgRNAs with 20 random guide RNA sequences and 3 bp NGG sequences (N can be A, T, G, or C) were designed to target the *CT001_A02071870* and *CT001_A06214910* genes using CRISPR direct (http://crispr.dbcls.jp; accessed on 10 April 2019). The discovery of off-target cleavage sites was analyzed by applying all possible nucleotide sequence combinations to the NGG sequences of the candidate target sequences. sgRNA was selected as a sequence with as few similar sequences in the Chinese cabbage genome as possible, which showed adequate GC content and targeted the exonic region of a gene. The sgRNA targeting the *BrLFY* paralogs was named LFY_sgRNA. LFY_sgRNA was ligated to the pHAtC backbone using *Sac*I restriction enzyme, the AtU6 promoter, and scaffold sequence. Clones were confirmed using the BTSeq™ sequencing service (Celemics, Seoul, Republic of Korea). The constructed gene-editing vector was transferred into *Agrobacterium tumefaciens* LBA4404 and used for Chinese cabbage transformation [46].

### 4.2. Production of the LFY-Edited Chinese Cabbage Lines and Selection Using PCR Analysis

*Agrobacterium*-mediated transformation of Chinese cabbage was performed [47]. The seeds of the inbred line, ‘CT001’, were cultured in MS basal medium in vitro and dark-treated for hypocotyl elongation. After two weeks of hygromycin selection pressure covering the transient expression period of *Agrobacterium*, the callus was transferred to a selective medium containing a selection marker. Notably, tentative T-DNA-inserted plants could grow and regenerate. Plants were cultivated in a controlled tissue-culture room at 20 °C under a 16 h light/8 h dark photoperiod.

Stable insertion of the T-DNA was detected via PCR analysis using the genomic DNA of ‘CT001’ and the tentative *LFY*-edited lines. Two sets of primers amplified the sections of the Hyg^R^ and Cas9hc regions present in the T-DNA. PCR amplification was performed with 2X H-Star *Taq* PCR master mix (Biofact, Seoul, Republic of Korea) in a total volume of 30 μL. The following reaction conditions were employed: initial denaturation at 95 °C for 15 min; 35 cycles of 95 °C for 30 s, 60 °C for 20 s, and 72 °C for 45 s; and a final extension step at 72 °C for 5 min. The PCR amplicons were electrophoresed on a 1% agarose.

### 4.3. Analysis of Mutagenesis and Expression Levels of the Target Gene

PCR and RT-PCR analysis was performed to identify the mutation events. The PCR amplicons were eluted using the P&C Multiple Elution Kit (Biosolution, Suwon, Republic of Korea) and sequenced using BTSeq™ sequencing service (Celemics, Seoul, Republic of Korea). The positive lines were sequenced to identify any mutations (insertion, deletion, nucleotide base transition, or transversion) within the target regions. The nucleotide sequence data obtained were aligned and translated into amino acid sequences. Comparisons with the inbred line, ‘CT001’, reference of the *BrLFY* paralogs were performed using the CLC sequence viewer software 8.0 (Qiagen, Aarhus, Denmark).

The expression levels of *BrLFY* paralogs were compared between the inbred line ‘CT001’ and E_0_ *LFY*-edited lines (LFY-7 and LFY-13) grown under long-day (16 h light/8 h dark) photoperiodic conditions. Total RNA was extracted from frozen tissue using the P&C Rapid RNA Prep Kit (Biosolution, Suwon, Republic of Korea). cDNA was synthesized from each sample using BioFACT™ RT-Kit (Biofact, Seoul, Republic of Korea). Each qPCR reaction contained 1 μL of cDNA, 2 μL of each primer, 5 μL of BioFACT™ 2X Real-Time PCR Master Mix (Biofact, Seoul, Republic of Korea), and water to a final volume of 10 μL. The qPCR cycling conditions began with a denaturing phase at 95 °C for 15 min; 35 cycles of 95 °C for 20 s, 55 °C for 20 s, and 72 °C for 20 s; and a final extension step at 72 °C for 5 min. The expression levels of *BrLFY* paralogs were normalized using the *BrActin7* gene, and each reaction was performed with three biological replicates. The relative expression levels of E_0_ *LFY*-edited lines were calculated using the 2^−ΔΔCt^ method.

### 4.4. Bolting Time Observation of the Inbred Line, ‘CT001’, and LFY-Edited Lines

To characterize the bolting time of the *LFY*-edited lines, ‘CT001’ and *LFY*-edited lines were artificially treated for 8 weeks in a cold room at 4 °C under 16 h/8 h (light/dark) photoperiod to induce a transition to the reproductive growth phase. The pots planted with ‘CT001’ and the *LFY*-edited lines were transferred to a greenhouse at 23 °C after cold-treatment. The status of bolting occurrence on a specific date was compared. After vernalization, the days of bolting and the first floral axis observations were recorded. The growth stages of the individual lines were observed at regular intervals. The selected *LFY*-edited lines were then advanced to the next generation.

### 4.5. Identification of the Number of T-DNA Copies and Insertion Position

Southern hybridization was performed to confirm the T-DNA inserted Chinese cabbage genome. A restriction enzyme with as many cleavage sites as possible in the inbred line ‘CT001’ genome was investigated. Genomic DNA was cut using the *Eco*RI restriction enzyme, which has approximately 45,000 cleavage sites in the genome. Although the *Eco*RI restriction enzyme has several cleavage sites in the genome, it is not present in the probe sequence. Therefore, normal hybridization was expected to occur. To predict the approximate molecular size of the product in which the hybridization signal occurred, lambda-*Hin*dIII molecular markers were loaded together. DNA fragments were separated overnight on a 1.0% agarose gel at 20 V and transferred to a Hybond N^+^ nylon membrane (Amersham Pharmacia, Buckinghamshire, UK). The membrane was UV-crosslinked and hybridized with a hybromycin DNA fragment with α-^32^P-labeled dCTP using BcaBEST labeling (TaKaRa, Otsu, Japan). The hybrid membrane was washed twice in a shaking incubator and exposed to an X-ray film in a cassette for 7 days.

To determine the T-DNA insertion site of the E_0_ *LFY*-edited lines, an effective variable argument thermal asymmetric interlaced PCR (VA-TAIL PCR) analysis was used [48]. The developed Chinese cabbage-specific AD primers and T-DNA-border-specific amplifying primer sets were used to amplify the junction region of T-DNA introduced into the *LFY*-edited line (Table S2 in Supplementary Materials). VA-TAIL PCR analysis was performed using 2X *Taq* PCR Master Mix (Biofact, Seoul, Republic of Korea) in a thermocycler (Applied Biosystems, Carlsbad, CA, USA). The total reaction volume was 30 μL and comprised 100 ng gDNA, AD, border-specific primers, and dH_2_O. The VA-TAIL PCR method and program conditions used in this study were the same as described in a previous study [49]. Specific amplicons were eluted using a P&C Multiple Elution Kit (Biosolution, Suwon, Republic of Korea), and the elution products were sequenced using the BTSeq^TM^ Contiguous Sequencing Service (Celemics, Seoul, Republic of Korea). Finally, the T-DNA insertion position (intergenic, exon, intron, 5′ upstream-1000, and 3′ downstream-300) was analyzed using the obtained sequence of the junction region.

## 5. Conclusions

*LEAFY* (*LFY*) is a transcription factor that determines floral identity and acts as a flowering inducer by interacting with various flowering-related genes. The *BrLFY* gene was structuralized and sgRNA was designed for CRISPR/Cas9 gene-editing vector construction. *Agrobacterium*-mediated transformation of Chinese cabbage plants was performed. By analyzing the characteristics of the selected *LFY*-edited lines, the bolting time was found to be delayed, even after the vernalization. CRISPR/Cas9-mediated mutagenesis of *BrLEAFY* disrupted the normal formation of amino acids, which delayed the bolting and flowering of Chinese cabbage. The transgene-free *LFY*-edited lines displayed the inheritance of base mutations and late bolting. These findings demonstrate that the CRISPR/Cas9 technology is an effective tool for mutagenesis in Chinese cabbage and that the *BrLFY* gene mutation influences the bolting time. Overall, to our knowledge, this is the first study to achieve effective *BrLFY* gene editing using the CRISPR/Cas9 system.

## 6. Patents

A patent applicated in Korea (patent application number 10-2021-0143792; application date 21 September 2021) with the following titles; “Gene-editing vector for *Brassica rapa* plant with late flowering trait and transformation method using the same”.

## Data Availability

Not applicable.

## Supplementary Materials

The supporting information can be downloaded at: https://www.mdpi.com/article/10.3390/ijms24010541/s1.
