# Supplementary material for: CRISPR/Cas9-Mediated Mutagenesis of BrLEAFY Delays the Bolting Time in Chinese Cabbage (Brassica rapa L. ssp. pekinensis)
Source: Int J Mol Sci. 2022 Dec 29;24(1):541. doi: 10.3390/ijms24010541 (PMC9820718; doi:10.3390/ijms24010541)
Supplement: Supplementary file 1 [file ijms-24-00541-s001.zip › ijms-2092790-supplementary.pdf]

(A)

	G	A	A	C	G	T	T	G	G	G	T	C	G	T	G	G	C		G	T	C	A	G	G
LFY-1	G	A	A	C	G	T	T	G	G	G	T	C	G	T	G	G	C		G	T	C	A	G	G
LFY-2	G	A	A	C	G	T	T	G	G	G	T	C	G	T	G	G	C	A	G	T	C	A	G	G
LFY-4	G	A	A	C	G	T	T	G	G	G	T	C	G	T	G	G	C	A	G	T	C	A	G	G
LFY-5	G	A	A	C	G	T	T	G	G	G	T	C	G	T	G	G	C		G	T	C	A	G	G
LFY-6	C	A	A	C	G	T	T	T	G	T	T	C	G	T	G	G	C		G	T	C	A	G	G
LFY-7	G	A	A	C	G	T	T	G	G	G	T	C	G	T	G	G	C	A	G	T	C	A	G	G
LFY-11	G	A	A	C	G	T	T	G	G	G	T	C	G	T	G	G	C		G	T	C	A	G	G
LFY-13	G	A	A	C	G	T	T	G	G	G	T	C	G	T	G	G	C	A	G	T	C	A	G	G
LFY-14	G	A	A	C	G	T	T	G	G	G	T	C	G	T	G	G	C		G	T	C	A	G	G

(B)

	G	A	A	C	G	T	T	G	G	G	T	C	G	T	G	G	C		G	T	C	A	G	G
LFY-1	G	A	A	C	G	T	T	G	G	G	T	C	G	T	G	G	C	T	G	T	C	A	G	G
LFY-2	G	A	A	C	G	T	T	G	G	G	T	C	G	T	G	G	C		G	T	C	A	G	G
LFY-4	G	A	A	C	G	T	T	G	G	G	T	C	G	T	G	G	C		G	T	C	A	G	G
LFY-5	G	A	A	C	G	T	T	G	G	G	T	C	G	T	G	G	C		G	T	C	A	G	G
LFY-6	G	A	A	C	G	T	T	G	G	G	T	C	G	T	G	G	C		G	T	C	A	G	G
LFY-7	G	A	A	C	G	T	T	G	G	G	T	C	G	T	G	G	C	A	G	T	C	A	G	G
LFY-11	G	A	A	C	G	T	T	G	G	G	T	C	G	T	G	G	C		G	T	C	C	G	G
LFY-13	G	A	A	C	G	T	T	G	G	G	T	C	G	T	G	G	C	C	G	T	C	A	G	G
LFY-14	G	A	A	C	G	T	T	G	G	G	T	C	G	T	G	G	C		G	T	C	A	G	G

Figure S1. Sequence-based detection of mutations induced by CRISPR/Cas9 system in the *BrLFY* paralogs in the E_0 *LFY*-edited lines. (A) Mutagenesis of *CT001_A02071870* at the genomic DNA level in the E_0 *LFY*-edited lines. (B) Mutagenesis of *CT001_A06214910* at the genomic DNA level in the E_0 *LFY*-edited lines. sgRNA sequence is underlined in black and blue rectangle indicates PAM sequence. Nucleotide mutations are indicated using red font.

(A) **(B)**

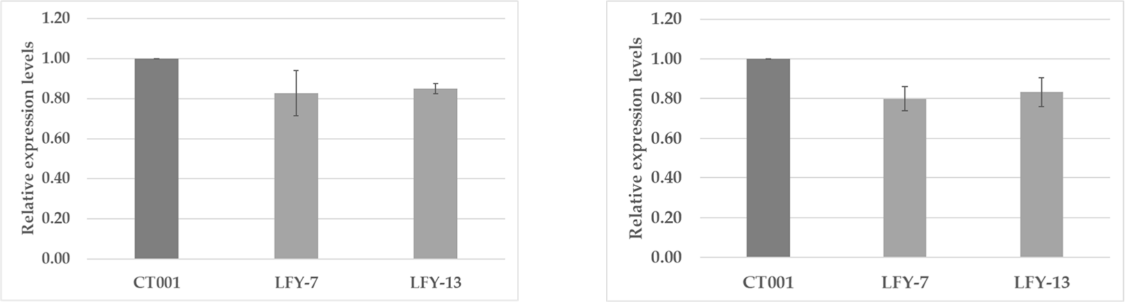

CT001_A02071870 ***CT001_ A06214910***

Figure S2. Comparison of the *BrLFY* paralogs expression levels between the inbred line 'CT001' and E_0 *LFY*-edited lines. (**A**) *CT001_A02071870* expression levels in the LFY-7 and LFY-13 edited lines. (**B**) *CT001_ A06214910* expression levels in the LFY-7 and LFY-13 edited lines. Values are averages from three independent experiments; the bars on the graph indicate the standard error of the means (n = 3). *BrActin7* gene was used as the endogenous housekeeping gene for normalization.

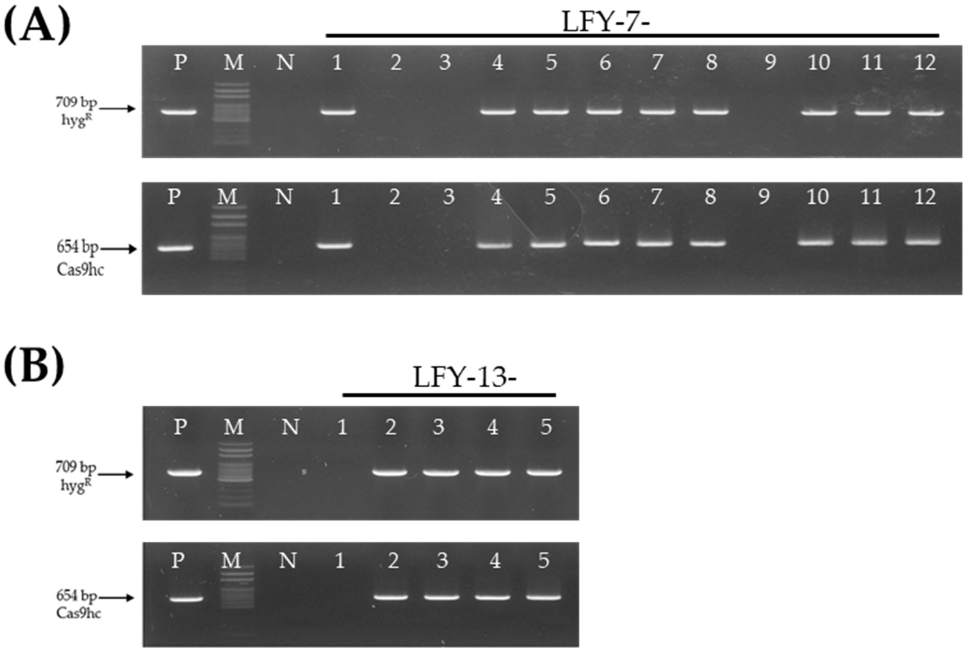

Figure S3. Identification of the E_1 *LFY*-edited lines using polymerase chain reaction (PCR) analysis. (**A**) PCR analysis with Hyg^R and Cas9hc primer sets of E_1 LFY-7-edited lines. (**B**) PCR analysis with Hyg^R and Cas9hc primer sets of E_1 LFY-13-edited lines. The 709 bp and 654 bp expected PCR products are indicated with an arrow, respectively. P, positive control; M, 100 bp DNA ladder; N, negative control; Numbering lane, *LFY*-edited lines.

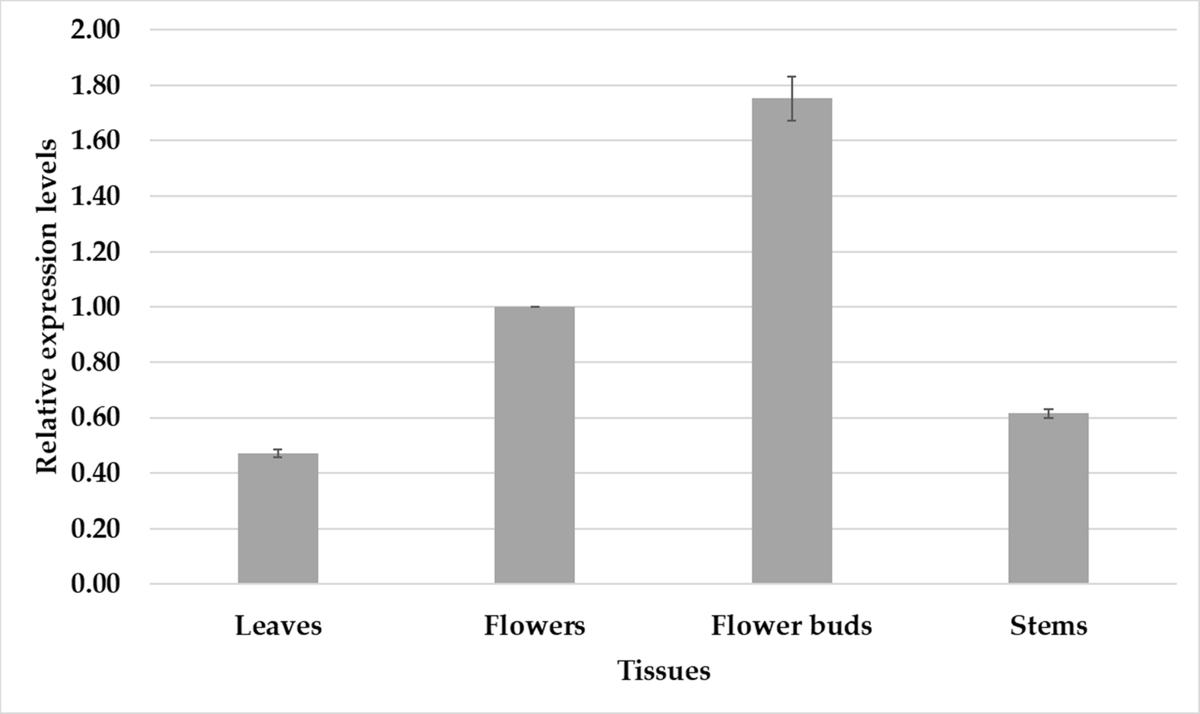

Figure S4. Expression levels of *CT001_A06214910* gene in different tissues of the inbred line 'CT001.' Based on the expression level of *CT001_A06214910* quantified in flowers, the relative expression levels of *CT001_A06214910* gene in leaves, flower buds, and stems were shown. Values are averages from three independent experiments; the bars on the graph indicate the standard error of the means (n = 3). *BrActin7* gene was used as the endogenous housekeeping gene for normalization.

Table S1. List of primer sets for polymerase chain reaction (PCR) analysis.

Name	Primer	Sequence (5'→3')	Expected product size (bp)
**Hyg^R**	F[z]	CGT CTG CTG CTC CAT ACA AG	709
	R	TGT CGA GAA GTT TCT GAT CGA	
**Cas9hc**	F	CCG CCA GGA GGA CTT CTA CC	654
	R	ATG TTC TCG GGC TTG TGG CG	
**CT001_A06214910**	F	CCA TAG TAC AGT ATC CAT GA	488
	R	GCG AGG ATG AGC GTT AAA	
**CT001_A020718710**	F	TGG TAC TTT CTG TTT GGA G	412
	R	TAC CGT TCC CAA CCA AAG	

[z]: F, forward primer; R, reverse primer.

Table S2. List of degenerate primers and T-DNA-border specific primers for variable argument-thermal asymmetric interlaced polymerase chain reaction (VA-TAIL PCR) analysis.

Target Region	Name	Sequence (5'→3')
**Zinc finger protein LSD1**	**BrAD1**[z]	GAM RTG NCT VAM WTT G[y]
	**BrAD2**	DTA ASA TGN HNT TGC T
**Left border of T-DNA**	**LSP1**	ATA GTG GAA ACC GAC GCC CCA GCA[x]
	**LSP2**	CCA GCA CTC GTC CGA GGG CAA AG
**Right border of T-DNA**	**RSP1**	AAA GTA TAC CCC TAC GAC GTG CCC G
	**RSP2**	GCC CGA CTA CGC CTA ACA CCC AG

[z] : BrAD primers are degenerate primers.

[y] : Mixed bases. M = A/C; R = A/G; V : A/C/G; W : A/T; D = A/G/T; S : G/C; H = A/C/T; N = any base.

[x] : Underlined nucleotide sequences indicate the overlapping sequence.
